# Prevalence of premature ovarian insufficiency and its determinants in Iranian populations: Tehran lipid and glucose study

**DOI:** 10.1186/s12905-021-01228-1

**Published:** 2021-02-23

**Authors:** Marzieh Rostami Dovom, Razieh ‌Bidhendi-Yarandi, Kazem Mohammad, Maryam Farahmand, Fereidoun Azizi, Fahimeh Ramezani Tehrani

**Affiliations:** 1 Reproductive Endocrinology Research Center, Research Institute for Endocrine Sciences, Shahid Beheshti University of Medical Sciences, No 24, Shahid Arabi St., Yaman Ave, Velenjak, P.O.Box, 19395-4763 Tehran, Iran; 2 Department of Epidemiology and Biostatistics, School of Public Health, Tehran University of Medical Sciences, Tehran, Iran; 3 Endocrine Research Center, Research Institute for Endocrine Sciences, Shahid Beheshti University of Medical Sciences, Tehran, Iran

**Keywords:** Premature ovarian failure, Premature ovarian insufficiency, Prevalence, Early menopause, Late menopause

## Abstract

**Background:**

Premature ovarian insufficiency (POI) considered as a concerning health issue for women of reproductive age. In this study we aim to estimate the prevalence of POI and assessing the influential factors.

**Methods:**

Data was obtained from Tehran lipid and glucose study (TLGS). All eligible post-menarcheal female participants of the TLGS, ages 20–65, were recruited (n = 6521). Participants were followed for the event of menopause, and age at menopause was recorded. Kaplan Meier analysis was applied to estimate mean and median for age at menopause. Weibull accelerated failure time survival regression model (AFT), was applied to assess influential determinants of POI. Conditional probability approach was used to provide estimation for prevalence of POI.

**Results:**

In this population-based study, the prevalence of POI (menopause age < 40 years) and early menopause (menopause age < 45 years) were estimated 3.5% and 24.6%, respectively. AFT model showed that in comparison to normal weight women, time to menopause was decreased by − 0.09 year (95% CI − 0.27, − 0.01, *p* = 0.023) and − 0.03 year (95% CI − 0.05, − 0.02, *p* = 0.000) in underweight and overweight women, respectively. Moreover, time to natural menopause was increased by 0.12 year (95% CI 0.07 to 0.17, *p* = 0.000) in women used oral contraceptives for > 6 months.

**Conclusion:**

About one quartile of Iranian women experienced menopause at an age less than 45, especially the non-normal weight ones; this high prevalence is a critical public health concerns that needs to be addressed by health policy makers.

**Supplementary Information:**

The online version contains supplementary material available at 10.1186/s12905-021-01228-1.

## Background

Menopause, a natural transitional process in women’s lives, usually occurs between the ages of 49 and 52 years [1]; about 4.2–7.6% of women experience menopause at ages < 45 [2, 3]. Early and late menopause are defined as menopause at 40–45 and > 55 years of age, respectively [4].

These conditions are associated with higher risk of cardiovascular disease, osteoporosis, and breast and endometrial cancers [5–7]. Premature ovarian insufficiency (POI) is defined as the cessation of menstruation along with serum estrogen deficiency and rising follicle-stimulating hormones (FSH) > 40 mIU/L at age < 40 [8]. Few studies have reported the prevalence of POI using different approaches; reporting a wide range for its prevalence from 1 to 5.5% [9–12]. In one approach, researchers included both menopausal and non-menopausal women and calculated the prevalence of POI by dividing the number of observed POI in the study population to the total number of married participants [11]. In another approach, participants were restricted to menopausal women [9] and the percentage of women with age at menopause < 40 was reported as the prevalence of POI; this restriction highly influenced the results and may have led to an increased risk of bias in the reported prevalence. To the best of our knowledge, no study has investigated the prevalence of POI using the first approach in the Iranian general populations. In the present study, we provide an estimation for POI, using data collected in the Tehran Lipid and Glucose Study (TLGS) and explore the risk factors among the general population of Iranian women.

## Methods

### Study population

For purposes of the present study, we used data collected in the Tehran Lipid and Glucose Study (TLGS); a cohort study initiated in 1998 to identify the risk factors for non-communicable diseases in Tehran urban populations. The detail of this population based study was reported elsewhere [13]. In brief, using a stratified cluster random sampling technique, 15,005 people aged ≥ 3 years were selected from the urban District 13 of Tehran, the capital of the Islamic Republic of Iran. The cohort members were physically examined every 3 years, and their demographic and anthropometric characteristics (education, marital status, and body mass index), smoking habits and reproductive characteristics (menarcheal age, number of pregnancy, abortions, delivery, contraception, and menopausal status) were assessed by trained interviewers during face-to-face interviews using a valid and reliable questionnaire (Additonal file 1 ). For the purpose of the present study, after excluding women who had reached menopause before initiation of the study (n = 1370), women had experienced non-natural menopause (medication, radiotherapy, surgery) (n = 600); those with uncertainty regarding their menopausal status or those within a transitional period of menopause (n = 931), there were 6521 post-menarcheal participants, ages 20–65, who were recruited for the present study. Menopause was defined according to the World Health Organization classification as a condition of absence of spontaneous menstrual bleeding for > 12 months for which no other pathologic or physiologic cause could be determined [14]. The time point of 1 year before the 12-month period of no menstrual bleeding was regarded as date of menopause and age at natural menopause (ANM) was calculated based on this time point. POI was defined as menopause before age 40 years.

The study protocol was approved by the Medical Ethics Committee of the Research Institute for Endocrine Sciences of Shahid Beheshti University of Medical Sciences and written informed consent was obtained from all participants.

### Statistical study

Baseline characteristics of the population were illustrated through descriptive statistics, and we calculated mean (SD) for normal and Median (IQR) for non-normal factors. To estimate the prevalence of POI, conditional probability of menopause event by age was used. We categorized age into 5 groups with 5-year intervals and then estimate the incidence of menopause in each age group, so in this way, cumulative distribution of age at menopause were calculated [15]. We conducted a Weibull accelerated failure time (AFT) survival regression model to investigate factors associated with ANM. AFT is a parametric survival model that regresses the logarithm of the survival time over the factors [16, 17]. Factors explored were included age at menarche, age at marriage, parity, abortion, gravity, history of menstrual cycle irregularity, smoking habits, obesity status and using OC for more than 6 months. Kaplan–Meier analysis was applied to estimate mean and median for ANM as well. Statistical analysis was done using STATA software (version 10; Stata Corp, College Station, TX, USA).

## Results

**Figure1:**
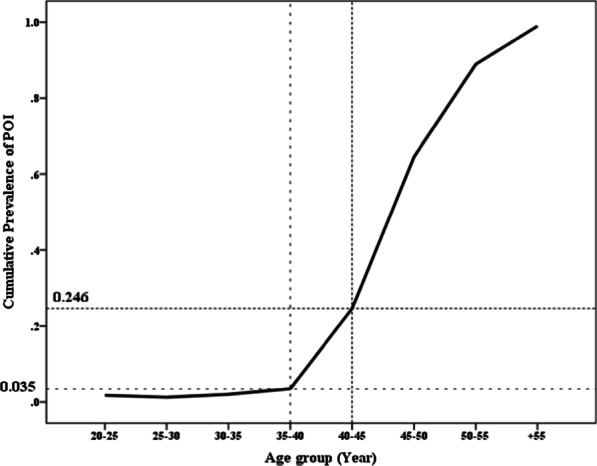
Cumulative distribution of age at natural menopause according to age groups

The characteristics of the study participants according to their menopausal status at the end of study is presented in Table 1. Mean (SD) age and BMI in menopause and non-menopause group were 56 (8) and 37(10) years, 28.8(5.4), and 27.1(6.03) kg/m2, respectively. Table 2 shows the cumulative distribution of onset of natural menopause in age groups; the prevalence of menopause for women age < 40, 45, 50, and 55 years were 3.5%, 24.6%, 64.5% and 88.9%, respectively. In addition, late menopause (> 55 years) was estimated approximately 1% (Table 2, Fig. 1). The mean and median (95% CI) for ANM using Kaplan–Meier analysis were 50.08 (95% CI 49.9, 50.3) and 51 (95% CI 50.8, 51.2), respectively. The age-adjusted AFT survival model revealed a significant positive association between OC use (> 6 months) and ANM ((0.12 (95% CI 0.07 to 0.17, *p* <  = 0.001)); a negative association between BMI − 0.001 (95% CI − 0.004 to − 0.001, *p* = 0.041) and ANM was observed (Table 3). Those with BMI < 18.5 kg/m^2^ and those with BMI ≥ 25 kg/m^2^ had shorter ANM compared to those with BMI 18.5–25 kg/m^2^ by − 0.09 (95% CI − 0.27, − 0.01, *p* = 0.023) and − 0.03 (95% CI − 0.05 to − 0.02, *p* < 0.001), respectively; we found no significant association between all other explored factors with ANM (Table 3).

**Table 1:** Characteristics of the study participants according to the menopausal status

	Menopause		
	No (n = 4605)	Yes (n = 1916)	*P-Value
Age(yrs.), mean (SD)	37(10)	56(8)	0.000*
Age of menarche(yrs.), Median (IQR)	13(1)	14(1)	0.000*
No abortion, Median (IQR)	1(1)	1(1)	0.954
No Birth, Median (IQR)	2(1)	4(2)	0.000*
BMI(kg/m2) Median (IQR)	27.1(6.03)	28.8(5.4)	0.000*
Menstrual irregularity, (%)	35.9	24.8	0.000*
OCs > 6 months, (%)	27	71	0.000*
Marital status, (%)			
Single	17	3.2	
Married	79.7	30.2	0.000*
Widow/divorced	3.3	66.6	
Smoking			
Daily	2.9	2.9	
Occasionally	2.5	0.8	0.000*
Never	94.6	96.3	
Education, (%)			
Illiterate	1.4	22.7	
< 5 years	14	4.6	
≥ 5 years	84.6	72.7	0.000*

BMI; body mass index, OC; oral contraceptives

*Obtained from *t* test for normal distribution, Mann Whitney for non-normal, and chi-squared for categorical variables

**Table 2:** Cumulative distribution of natural menopause by age group

	Menopause	
	No, n (%)	Yes, n (%)
Age group (years)		
20–25	1180 (98.3%)	21 (1.7%)
25–30	955 (98.8%)	12 (1.2%)
30–35	928 (98.0%)	19 (2.0%)
35–40	663 (96.5%)	24 (3.5%)*
40–45	608 (75.4%)	198 (24.6%)
45–50	201 (35.5%)	365 (64.5%)
50–55	62 (11.1%)	497 (88.9%)
+ 55	8 (1.0%)	780 (99.0%)

*Cumulative prevalence of primary ovarian insufficiency (POI) defined as menopause before age 40 years

**Table3:** Results of age-adjusted Weibull accelerated failure time (AFT) survival regression model to explore potential factors associated with age at natural menopause

Factors	Parametric survival	*p *Value
	regression coefficient (95% CI)	
Age of menarche(years)	0.00 (− 0.01, 0.01)	0.951
Age at marriage	− 0.09 (− 0.43, 0.23)	0.524
Gravity	− 0.11 (− 0.46, 0.23)	0.506
Number of abortions	− 0.01 (− 0.02, 0.01)	0.405
Parity	0.00 (− 0.00, 0.01)	0.223
BMI		
Underweight < 18.5	− 0.09 (− 0.27, − 0.01)	0.023*
Overweight/Obese > 25	− 0.03 (− 0.05, − 0.02)	0.000*
Normal 18.5–24.9	Ref	–
Having history of irregular(yes/no) menstrual cycle	0.03 (− 0.01, 0.06)	0.135
Smoking habitus		
Daily	− 0.04 (− 0.10, 0.02)	0.205
Occasionally	0.00 (− 0.11, 0.13)	0.908
Never	–	–
OCs > 6 months (yes/no)	0.12 (0.07, 0.17)	0.001*

BMI; body mass index, OC; oral contraceptives

Weibull accelerated failure time survival regression model was run over 3560 non-menopause women who were followed to reach the event

*Significant level at < 0.05

## Discussion

In this study, we found that 3.5% of participants experienced POI, 24.6% experienced early menopause. Among all those assumed anthropometric and reproductive determinants of age at natural menopause, consumption of OC for > 6 months and BMI were influential factors that had significant positive and negative effects on ANM, respectively. The prevalence of POI is approximately 1% to 5.5% worldwide [18]. The differences in the prevalence of natural menopause in the literature could be due to methodological choices. Through a comprehensive systematic literature review, we found that different studies had conducted different approaches to estimate these measures, such as the prevalence of POI. For instance, some of the studies counted the number of POI cases among the total population and estimated the prevalence of POI, leading to underestimated measurements [2, 19]; other studies, which considered POI cases among natural or natural and medical menopause populations, provided overestimated and also biased measurements [20–38].We used logistic regression to estimate the cumulative prevalence of POI. By this approach, we were able to estimate unbiased estimates of the prevalence [39, 40]. In addition, the Accelerated Failure Time model was applied to investigate the factors that affected age at menopause. That approach regresses the logarithm of the survival time over the factors and provides a robust parametric model. AFT is very desirable since it is valid under weaker conditions than the Cox proportional hazard model [17]. The novelty of the present study is the methodological approach to POI prevalence estimation with minimal risk of bias in over- or underestimation. Applying the AFT model, we found that OC consumption and BMI were influential determinants of ANM. Contradictory results have been reported regarding the relationship between contraceptive pills and age of menopause [41–44]. In some studies oral contraceptive use were significantly associated with later age at menopause [41, 44], while Hassa et al. have no found any association between contraceptive use and age at menopause [42]. We found that those women who used OC for > 6 months experienced menopause on average one year (obtained from AFT formula model) later than non-users. Our findings concur with De-Vries et al.’s study [44], which found that each year’s use of OC was associated with an increase of 1.2 months of ANM. The main pathophysiologic aspect of this association has not been identified but it may be due to prevention of ovulation. Other studies have not reported this association [43, 45]. In Stanford et al., the association between the use of OC and age at menopause became non-significant after accounting for the time of exposure. The association between BMI and ANM seems to be nonlinear, as both underweight and overweight women experienced menopause earlier. The women with lower BMI (< 18.5 kg/m2) have poor fat storing that may lead to poor quality of ovarian follicles [46–48]. In contrast to the general perception, lower BMI is unrelated to a decline of estrogen biosynthesis in fatty tissue in lean women, because the onset of estrogen synthesis in peripheral fat happens during postmenopausal time [49]. The association between obesity and ANM may be partly explained through these two hypotheses: (1) In the general population, obesity is associated with hyperinsulinemia, insulin resistance, and steroid hormone binding globulin (SHBG) reduction, which elevates testosterone, dihydrotestosterone, and androstenediol [50]. Decreasing SHBG are related to increasing insulin, glucose, triglycerides, and C-reactive protein, an inflammatory marker in follicular fluid [51]. On the other hand, aging increases insulin resistance and the risk of diabetes type 2 per se. Insulin resistance has a close association with hyperinsulinemia [52]. Moreover, the negative association between the antimullerian hormone (AMH), a valuable marker of ovarian reserve, and Homeostatic Model Assessment for Insulin Resistance (HOMA-IR) levels have been reported [53]. Considering these associations may explain the decline of ANM in overweight/obese women (2). Ototoxicity of inflammatory factors secreted by adipose tissue may lead to poor quality of oocytes that have been observed in obese mice [54] and humans [55–57]. The relationship between elevated levels of C reactive protein and other oxidative stress factors and impaired oocyte development has been suggested by some studies [51, 58]. Theca cells in follicles need insulin for stimulating steroidogenesis and for upregulating luteinizing hormone (LH) receptors expression. Due to hyperinsulinemia in obese women, more receptors will be up regulated and the balance of LH to FSH ratio may disrupted, consequently ovulation and oocyte maturation will be interrupted [50, 59]. The main strength of our study is its methodology because we used data from a population-based cohort study of non-menopausal women and followed them for about two decades for the event of menopause. Additionally, we used a Weibull accelerated failure time survival regression model that is most fitted for data on ANM regarding the flexibility in modeling ANM based on the shape of probability distribution. We also conducted an AFT model to provide a robust inference. The weakness of this study may the recall bias for exact age at menopause, although three- year’s interval for follow-up seems to provide a precise estimation. The absence of FSH measurement is another weakness of this study. Larger longitudinal population-based studies starting with women in their early reproductive years and following them until menopause are needed for more precise estimation of ANM.

## Conclusion

About one quartile of Iranian women experienced menopause at an age less than 45, especially the non-normal weight ones; informing the women regarding the negative impact of having abnormal weight throughout the reproductive period, on the age at menopause is recommended.

## Supplementary Information

**Additional file 1** (PDF 400 KB)

## Data Availability

The dataset used and analyzed during the current study are available from the corresponding authors upon making official request.

## Abbreviations

POI: premature ovarian insufficiency
TLGS: Tehran lipid and glucose study
AFT: accelerated failure time survival regression model
BMI: body mass index
OC: oral contraceptive
ANM: age at natural menopause
