# Supplementary material for: Prevalence of premature ovarian insufficiency and its determinants in Iranian populations: Tehran lipid and glucose study
Source: BMC Womens Health. 2021 Feb 23;21:79. doi: 10.1186/s12905-021-01228-1 (PMC7903639; doi:10.1186/s12905-021-01228-1)
Supplement: Supplementary file 1 — Additional file 1 (PDF 400 KB) [file 12905_2021_1228_MOESM1_ESM.pdf]

C) Questions on personal habits:	
**1-Do you smoke cigarettes?**<br>1- Yes, regularly 2- No (go to question 3)<br>3- Yes, irregularly (go to question 3)	☐
**2-On the average , how many cigarettes do you smoke per day ?**<br>1) 1-5 2) 6-10 3) 11-15 4) 16-20 5) >20<br>6) insufficient data<br>Then go to question 5	☐
**3- Have you ever smoked cigarettes in the past?**<br>1- Yes, regularly 2- No (go to question 6)<br>3- Yes, irregularly (go to question 6)	☐
**4- How many years have you stopped smoking?**<br>(99- Don't know)	☐☐
**5- How old were you when you first started to smoke regularly?** (99- Don't know)	☐☐
**6- Do you smoke pipe now?**<br>1- Yes, regularly 2- No (go to question 9)<br>3- Yes, irregularly	☐
**7- How many days does a pack of pipe tobacco last?**<br>(99- Don't know)	☐☐

C) Questions on personal habis:

**8- How old were you when you first started to smoke pipe?**<br>(99- Don't know)	☐☐
**9- Have you ever smoked a pipe in the past?**<br>1- Yes, regularly 2- No<br>3- Yes, irregularly	☐
**10- Do you smoke hookah (ghalian) ?**<br>1- Yes, regularly 2- No (go to question 13)<br>3- Yes, irregularly	☐
**11- How many days does a pack of tobacco last for hookah (ghalian) ?** (99- Don't know)	☐☐
**12- How old were you when you first started to smoke hookah (ghalian) ?** (99- Don't know)<br>Then go to question 14.	☐☐
**13- Have you ever smoked hookah (ghalian) in the past?**<br>1- Yes, regularly 2- No 3- Yes, irregularly	☐

The following questions are asked from all age groups:

**14- Is there any cigarette smoker in your house that you inhale his/ her tobacco smoke?**<br>1- Yes 2- No	☐

E) The following questions are asked from women only:	
**1-Do you have normal menstrual cycles? (for cases>10years of age)**<br>1-Yes, naturally without taking control pills(go to question 5)<br>2- No (go to question 13)<br>3- Prepubertal age (go to physical exam section)<br>4- Yes, but with hormone replacement pills	☐
**2-Are you currently taking any hormone replacement medication?**<br>1- Estrogen 2- Progesterone 3-Both 4-None	☐
**3- For how long has your menstrual cycles stopped? (Cases >15 years of ages) (Month and year)**	***Year*** ☐☐<br>***Month*** ☐☐
**4- What is the reason of your menstrual cycles stopping?**<br>1- Natural menopause<br>2- Early-onset menopause<br>3- Hysterectomy without oophorectomy or unilateral oophorectomy<br>4- Hysterectomy + bilateral oophorectomy<br>5- Bilateral oophorectomy without hysterectomy<br>6- Other<br>7- Don’t know	☐

E) The following questions are for married women only:	
**5- Which contraception method have you used during the last three months?**<br>1-LD contraceptive 2-HD contraceptive<br>3-Minipill 4- Triphasic pill	☐
**6- Are you pregnant now?**<br>1-Yes 2-No 3-Don't know	☐
**7- Have you ever been pregnant?**<br>1- Yes 2-No (go to physical exam section)	☐
**8- How many live born children have you had?**	☐☐
**9- Have you given birth to a baby in the past one year?**<br>1- Yes 2-No	☐
**10- Have you ever had any abortion?**<br>1- Yes 2- No (go to question 14)	☐
**11- How many abortions have you had?**	☐
**12- Do you have any history of stillbirth?**<br>1- Yes 2- No (go to question 14)	☐

F) The following questions are for married women only:

**13- How many stillbirths have you ever experienced?**	☐☐
**14- Has a doctor ever told you that you had hypertension or convulsion during any of your pregnancies?**<br>1- Yes 2- No 3- Unknown	☐
**15- Has a doctor ever told you that you had high blood glucose or gestational diabetes during any of your pregnancies?**<br>1-Yes 2- No 3- Unknown	☐
**16- Have you ever had a baby that weighed 4.5 Kg or more?**<br>1-Yes 2- No 3- Unknown	☐
**17- Are you breast-feeding now?**<br>1-Yes 2- No 3- Unknown	☐

G) Physical Examination Summery:

**12-Anthropometry**<br>1- Height (cm)<br>2- Weight (kg)<br>3- Waist circumference (cm)<br>4- Hip circumference (cm)<br>5- Wrist circumference (cm with one decimal digit)	……………………………<br>……………………………<br>……………………………<br>……………………………<br>……………………………
